# Identification of gene networks jointly associated with depressive symptoms and cardiovascular health metrics using whole blood transcriptome in the Young Finns Study

**DOI:** 10.3389/fpsyt.2024.1345159

**Published:** 2024-04-25

**Authors:** Binisha H. Mishra, Emma Raitoharju, Nina Mononen, Aino Saarinen, Jorma Viikari, Markus Juonala, Nina Hutri-Kähönen, Mika Kähönen, Olli T. Raitakari, Terho Lehtimäki, Pashupati P. Mishra

**Affiliations:** ^1^Department of Clinical Chemistry, Faculty of Medicine and Health Technology, Tampere University, Tampere, Finland; ^2^Finnish Cardiovascular Research Center Tampere, Faculty of Medicine and Health Technology, Tampere University, Tampere, Finland; ^3^Department of Clinical Chemistry, Fimlab Laboratories, Tampere, Finland; ^4^Molecular Epidemiology, Faculty of Medicine and Health Technology, Tampere University, Tampere, Finland; ^5^Tampere University Hospital, Tampere, Finland; ^6^Department of Psychology and Logopedics, Faculty of Medicine, University of Helsinki. Helsinki, Finland; ^7^Helsinki University Central Hospital, Adolescent Psychiatry Outpatient Clinic, Helsinki, Finland; ^8^Department of Medicine, University of Turku, Turku, Finland; ^9^Division of Medicine, Turku University Hospital, Turku, Finland; ^10^Department of Paediatrics, Tampere University Hospital, Faculty of Medicine and Health Technology, Tampere University, Tampere, Finland; ^11^Department of Clinical Physiology, Tampere University Hospital, Tampere, Finland; ^12^Research Centre of Applied and Preventive Cardiovascular Medicine, University of Turku, Turku, Finland; ^13^Department of Clinical Physiology and Nuclear Medicine, Turku University Hospital, Turku, Finland; ^14^Centre for Population Health Research, University of Turku and Turku University Hospital, Turku, Finland

**Keywords:** depressive symptoms, cardiovascular health, comorbidity, multimorbidity, transcriptome, gene networks

## Abstract

**Background:**

Studies have shown that cardiovascular health (CVH) is related to depression. We aimed to identify gene networks jointly associated with depressive symptoms and cardiovascular health metrics using the whole blood transcriptome.

**Materials and methods:**

We analyzed human blood transcriptomic data to identify gene co-expression networks, termed gene modules, shared by Beck’s depression inventory (BDI-II) scores and cardiovascular health (CVH) metrics as markers of depression and cardiovascular health, respectively. The BDI-II scores were derived from Beck’s Depression Inventory, a 21-item self-report inventory that measures the characteristics and symptoms of depression. CVH metrics were defined according to the American Heart Association criteria using seven indices: smoking, diet, physical activity, body mass index (BMI), blood pressure, total cholesterol, and fasting glucose. Joint association of the modules, identified with weighted co-expression analysis, as well as the member genes of the modules with the markers of depression and CVH were tested with multivariate analysis of variance (MANOVA).

**Results:**

We identified a gene module with 256 genes that were significantly correlated with both the BDI-II score and CVH metrics. Based on the MANOVA test results adjusted for age and sex, the module was associated with both depression and CVH markers. The three most significant member genes in the module were *YOD1*, *RBX1*, and *LEPR*. Genes in the module were enriched with biological pathways involved in brain diseases such as Alzheimer’s, Parkinson’s, and Huntington’s.

**Conclusions:**

The identified gene module and its members can provide new joint biomarkers for depression and CVH.

## 1 Background

Depression and cardiovascular disease (CVD) are two key global public health issues with major human suffering and economic costs (1–3). Studies have shown that depression and CVD are associated (3–6). Previous studies have shown that while about 15% of patients with CVD have major depression (7), about two-thirds of patients hospitalized after acute myocardial infarction (AMI) have mild depression (8). In addition, a study by (9) suggests that early treatment for depression, before the development of symptomatic cardiovascular disease, can reduce the risk of CVD by almost half. Based on accumulating evidence, the American Heart Association (AHA) issued a statement in 2015 that clinicians should actively monitor teenage patients with depression and bipolar disorder because they have an increased risk for CVD (10). Therefore, as patients with CVD have an increased risk of developing depression (10) and people with depression have an increased risk for CVD (11), there appears to be a bidirectional relationship between the two conditions. It is, however, unclear whether CVD leads to depression or whether depression leads to CVD due to lifestyle factors that increase the risk for CVD or both. Therefore, as the two conditions can be either comorbid or multimorbid conditions (12), we refer these conditions as potential co/multimorbid conditions in the remainder of the study.

The elevated risk of CVD among people with depression could be at least partly due to lifestyle factors such as cigarette smoking, alcohol use, lack of exercise, unhealthy diet, and medications (13, 14). The biological processes involved in the pathophysiology of depression may also interfere with those linked to cardiovascular health, ultimately leading to CVD. For instance, depressed patients with high stress levels have increased glucocorticoid concentrations, which causes hyperglycemia and can eventually result in CVD (15, 16). The hypothalamic–pituitary–adrenal (HPA) axis, a stress response mechanism involving the central nervous system and endocrine system, increases the risk of CVD by promoting the development of dyslipidemia and hypertension (17). The HPA axis is also upregulated in patients with depression (18). Similarly, depletion of neurotransmitters, such as serotonin, dopamine, and norepinephrine is known to be involved in the pathophysiology of depression (19). These neurotransmitters have also been associated with CVD and its associated risk factors. For example, serotonin is associated with coronary artery disease and cardiac events (20). Dopamine plays an important role in the pathogenesis of hypertension (21). Similarly, norepinephrine acts on the vasavasorum, reducing the flow of oxygen, and ultimately resulting in hypoxia. The restricted oxygen supply plays a vital role throughout the atherosclerotic vulnerable plaque formation process (22). Inflammation, which is a significant contributor to the development and progression of atherosclerotic CVD (23), may also impair the function of these neurotransmitters (24). A chronic low-grade inflammatory response is associated with depression (25). Increased levels of inflammatory markers, such as serum C-reactive protein and interleukin-6, which play roles in CVD pathogenesis, are also known to be associated with depression (26). Beyond the factors that might account for the association between depression and CVD, the biological mechanisms underlying these potential co/multimorbid conditions seem complex and remain largely unknown. Given the burden of these conditions on patients, the development of targeted interventions for depression and CVD is crucial. This requires an understanding of the genetics and biological pathways shared by these two conditions, and the disentanglement of the bidirectional relationship between depression and CVD at the molecular level.

In the present study, we hypothesized that the complexity of the relationship between depression and cardiovascular health (CVH) can be, at least partly, explained by the underlying gene networks. Therefore, the objective of the present study was to identify transcriptomic networks jointly associated with markers of both depression and CVH using a system-level bioinformatics approach. We conducted weighted gene co-expression network analysis (WGCNA) (27) of whole blood transcriptomic data, followed by multivariate analysis of variance (MANOVA) and pathway analysis to identify gene co-expression networks, termed gene modules, enriched biological pathways in the modules, and the most important member genes jointly associated with markers of both depression and CVH. The analysis pipeline has been described in detail elsewhere (28, 29).

## 2 Materials and methods

### 2.1 Study participants

This study was based on the Young Finns Study (YFS), one of the largest prospective multicenter follow-up studies assessing cardiovascular risk factors from childhood to adulthood (30). The study began in 1980 with 3,596 children and adolescents aged 3–18 years, randomly selected from five university hospitals in Finland (Turku, Tampere, Helsinki, Kuopio, and Oulu). The participants were regularly followed for over 40 years. The study was approved by the ethics committee of the Hospital District of Southwest Finland on 20 June 2017 (ETMK:68/1801/2017). All participants provided written informed consent and the study was conducted in accordance with the Declaration of Helsinki. Data protection will be handled according to the current regulations. The present study was based on 899 participants (women: 59%), aged 34–49 years, from the 2011 follow-up (**Table 1**), with ideal CVH metrics as a marker of CVH, Beck’s depression inventory (BDI-II) score as a marker of depression, and transcriptomics data.

**Table 1:** Population characteristics of the Young Finns Study cohort.

	Men	Women
Number of subjects	370 (41%)	529 (59%)
Age, years	42 (±5)	42 (±5)
Body mass index, kg/m^2^	26.8(±4.4)	25.9 (±5.2)
Total cholesterol (mmol/l)	5.3 (±0.9)	5.1 (±0.9)
LDL cholesterol (mmol/l)	3.4 (±0.9)	3.1 (±0.8)
HDL cholesterol (mmol/l)	1.2 (±0.3)	1.4 (±0.3)
Triglycerides (mmol/l)	1.5 ( ± 1.5)	1.1 ( ± 1.6)
Serum glucose (mmol/l)	5.5 (±0.9)	5.2 (±0.7)
C-reactive protein (mg/l)	1.3 (±1.9)	1.7 (±2.5)
Systolic blood pressure (mmHg)	123 (± 13)	116 (±14)
Diastolic blood pressure (mmHg)	77 (±10)	73 (±9)
Alcohol consumption, units/day	1.1 (±1.3)	0.5(±0.7)
Physical activity index (MET-h/wk)	21.8 (±22.3)	21.3 (±19.3)
Daily smoking, %	47/370 (13%)	50/529 (10%)
Participants with type 1 diabetes (%)	1/359 (0.3%)	2/512 (0.4%)
Participants with type 2 diabetes (%)	12/369 (3%)	17/527 (3%)

Data are expressed as means (±SD) or proportions (%).

### 2.2 Cardiovascular health metrics

The American Heart Association (AHA) has defined ideal CVH as the simultaneous presence of four favorable health behaviors (nonsmoking, ideal body mass index (BMI), physical activity, and healthy diet) and three favorable health factors (ideal levels of total cholesterol, blood pressure, and fasting glucose) (31). We generated AHA indices for the seven corresponding health behaviors and factors. The ideal CVH metrics simply correspond to the number of ideal health factors and behaviors present during the follow-up period, as described elsewhere (32). The score ranges from 0 to 7, with seven being the best score representing ideal CVH.

### 2.3 Beck’s depression inventory (BDI) score

The BDI score used in this study was based on the revised version of BDI-II (33) of the original BDI (34). Like the original BDI, the BDI-II contains 21 questions measuring the characteristic attitudes and symptoms associated with depression. Each answer is scored on a scale of 0 to 3, with higher scores representing higher levels of depression. Instead of the past week, as in the original BDI, participants were asked to report how they had been feeling over the past two weeks.

### 2.4 Blood transcriptomics

Whole genome blood transcriptome was profiled from the RNA isolated from whole blood (2.5 ml) collected from YFS participants during the 2011 follow-up in PaXgene Blood RNA Tubes (PreAnalytix, Hombrechtikon, Switzerland). The tubes were inverted 8–10 times and stored at room temperature for at least 2 h. PaXgene tubes were frozen and stored less than one year in −80°C. After thawing, the tubes were stored at room temperature (2 h–12 h), according to the manufacturer’s instructions. RNA was then isolated using the PAXgene Blood RNA Kit (Qiagen) with DNase Set according to the manufacturer’s instructions using the QiaCube.

The concentration and purity of RNA samples were evaluated spectrophotometrically using a NanoDrop spectrophotometer (BioPhotomer, Eppendorf, Wesseling-Berzdorf, Germany). Samples were considered pure if the 260/280 ratio was in the range of 1.8–2.2. The RNA isolation process was validated by analyzing the integrity of the RNA using the RNA 6000 Nano Chip Kit (Agilent). The RNA integrity number (RIN) and shape of the electropherogram of the 26 RNA samples isolated from YFS were evaluated. Visual inspection of the shape of the electropherogram revealed no samples with signs of degradation. The average RIN value was 8.2 with a standard deviation of 0.5.

Expression levels were analyzed using the Illumina HumanHT-12 version 4 Expression BeadChip (Illumina Inc.) containing 47,231 expression and 770 control probes. Briefly, 200 ng of RNA was reverse transcribed into cDNA and biotin-UTP labeled using the Illumina TotalPrep RNA Amplification Kit (Ambion). Next, 1,500 ng of cDNA was hybridized to the Illumina HumanHT-12 v4 Expression BeadChip. BeadChips were scanned using an Illumina iScan system. Samples with fewer than 6,000 significantly detected expression probes (detection p-value <0.01) were discarded. Raw Illumina summary probe-level data were exported from Beadstudio and processed in R (http://www.r-project.org/) using nonparametric background correction, followed by quantile normalization with control and expression probes, with the *neqc* function in the limma package (35) and a log2 transformation. Samples with a sex mismatch between the recorded sex and predicted sex based on *RPS4Y1-2* and *XIST* mRNA levels on the Y and X chromosomes, respectively, were excluded.

### 2.5 Biostatistical analysis

**Figure 1:**
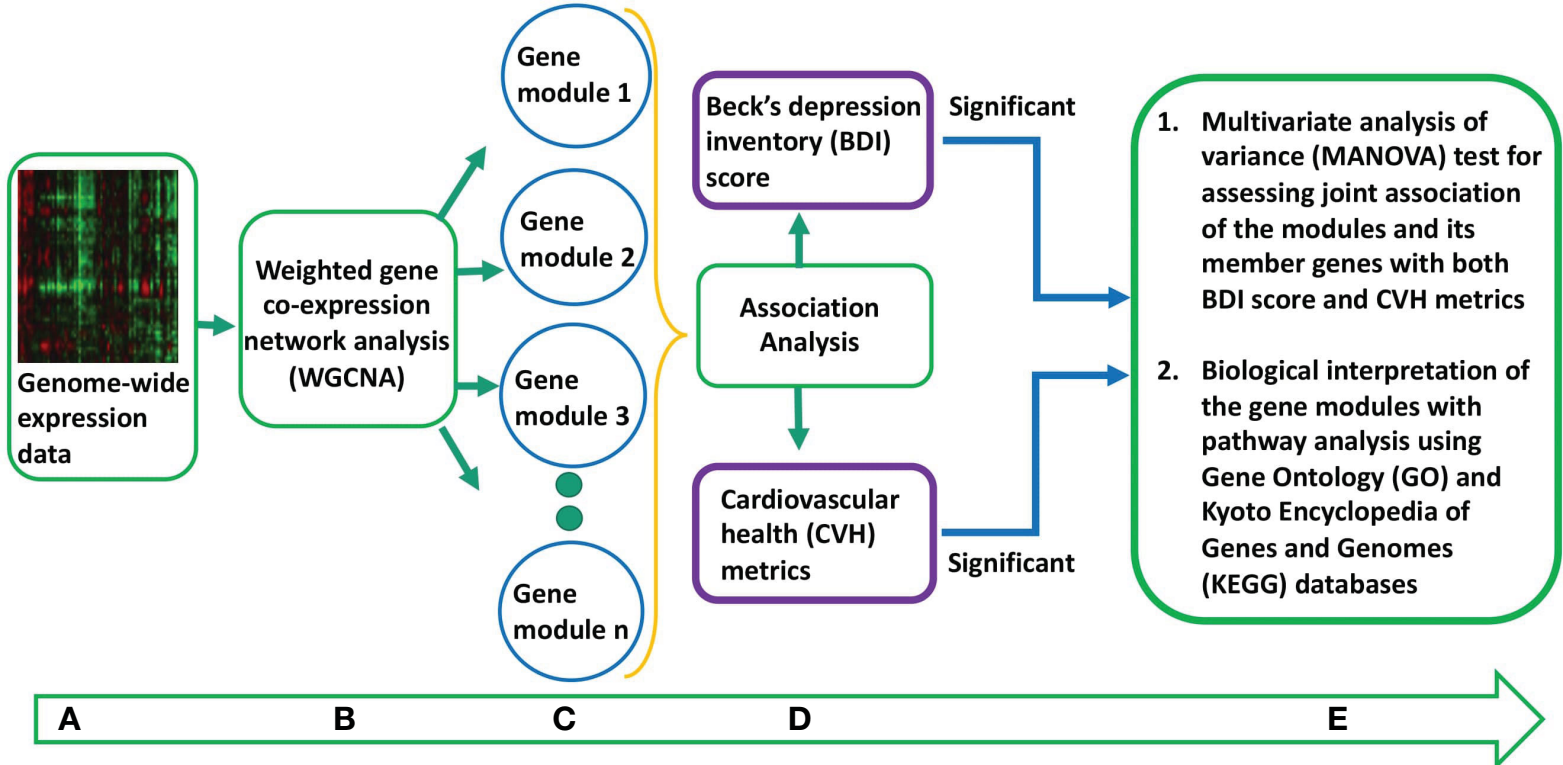
Overall concept of the co/multimorbidity analysis pipeline. **(A)** Pre-processed genome-wide expression data. **(B)** Weighted gene co-expression gene network analysis to identify gene co-expression networks or modules. **(C)** Gene modules identified from step **(B)**. **(D)** Association analysis of the eigengenes of the gene modules with markers of both depression and cardiovascular health (CVH). The gene modules that are significantly associated with markers of both depression and CVH are considered as candidate gene modules for co/multimorbidity study. **(E)** Multivariate analysis of variance (MANOVA) test to access joint association of the candidate gene modules as well as its member genes with the markers of both depression and CVH. Biological interpretation of the jointly associated gene modules is done by pathway enrichment analysis of the genes in the modules.

All statistical analyses and data processing were performed using the statistical package R version 3.6.0 (36). The overall concept of the co/multimorbidity analysis involved i) identification of gene modules with densely interconnected genes, ii) association of the identified gene modules with markers of both depression and CVH independently, iii) MANOVA test to assess the joint association of the gene modules as well as its member genes with the markers of both depression and CVH, and iv) biological interpretation of the jointly associated gene modules with pathway enrichment analysis of the genes in the modules (**Figure 1**). The ideal CVH metrics from YFS participants were normally distributed (**Supplementary Figure S2**). The BDI-II scores were log-transformed to correct for skewness (**Supplementary Figure S3**).

Gene modules, groups of densely interconnected genes, were identified using *WGCNA* implemented with R statistical software (27). Gene module generation with *WGCNA* involved multiple steps. The first step involved the calculation of Pearson’s correlation (r) for all pairwise comparisons of genes across all participants, resulting in a correlation matrix. The correlation matrix was then raised to a power of five, chosen using the power function implemented in the WGCNA package, to generate an adjacency matrix to minimize noise and emphasize stronger correlations (**Supplementary Figure S1**). This approach transforms the correlation matrix into an approximately scale-free topology based on the assumption that most real-world biological networks are scale-free. Next, a topological overlap matrix (TOM), a similarity matrix of genes, was generated from the resulting adjacency matrix to incorporate network topology information into the definition of gene co-expression. TOM was transformed into a dissimilarity matrix (1-TOM). A hierarchical clustering tree of the genes was then generated by average linkage-based hierarchical clustering of the dissimilarity matrix. Gene modules from the hierarchical clusters of genes were identified using a dynamic tree-cutting algorithm. We assessed the quality of the identified gene modules by analyzing the correlation between gene significance (GS) and module membership (MM). GS is defined as the correlation between the module’s individual member gene expression levels and markers of both CVH and depression (ideal CVH metrics and BDI-II score, respectively). MM is defined as the correlation between the summary expression profile of a module (the first principal component, termed the eigengene of the module) and expression levels of its member genes. An ideal gene module is one where GS and MM are highly correlated, suggesting that the genes that are highly correlated with the studied markers are also important members of the analyzed module.

The first principal component of the expression profiles of the member genes in a module, termed the eigengene, was used as a surrogate for the summary expression profile of the module. Pearson’s correlation coefficients (r) were calculated between the eigengenes of the identified gene modules and markers of CVH and depression. Gene modules that were significantly correlated (p-value <0.05) with markers of both CVH and depression were considered candidate modules for testing the joint statistical association with the studied markers of both CVH and depression in multivariate statistical analysis. The joint association of the candidate gene modules with CVH and depression markers were tested using the MANOVA test implemented in the *car* R package. MANOVA was adjusted for age and sex. Pathway enrichment analysis of the genes in the significant gene modules was performed using biological process Gene Ontology (GO) terms (37) and the Kyoto Encyclopedia of Genes and Genomes (KEGG) (38) using *clusterProfiler* R/Bioconductor packages (39). The analysis pipeline has been described elsewhere (28).

## 3 Results

### 3.1 Study population characteristics

#### 3.2 Association between the markers of cardiovascular health and depression

The CVH metrics had a weak but significant negative correlation (*r* = −0.10, *p-*value = 0.01) with the BDI-II score (**Supplementary Figure S4**).

#### 3.3 Association between the identified gene modules and markers of cardiovascular health and depression

**Figure 2:**
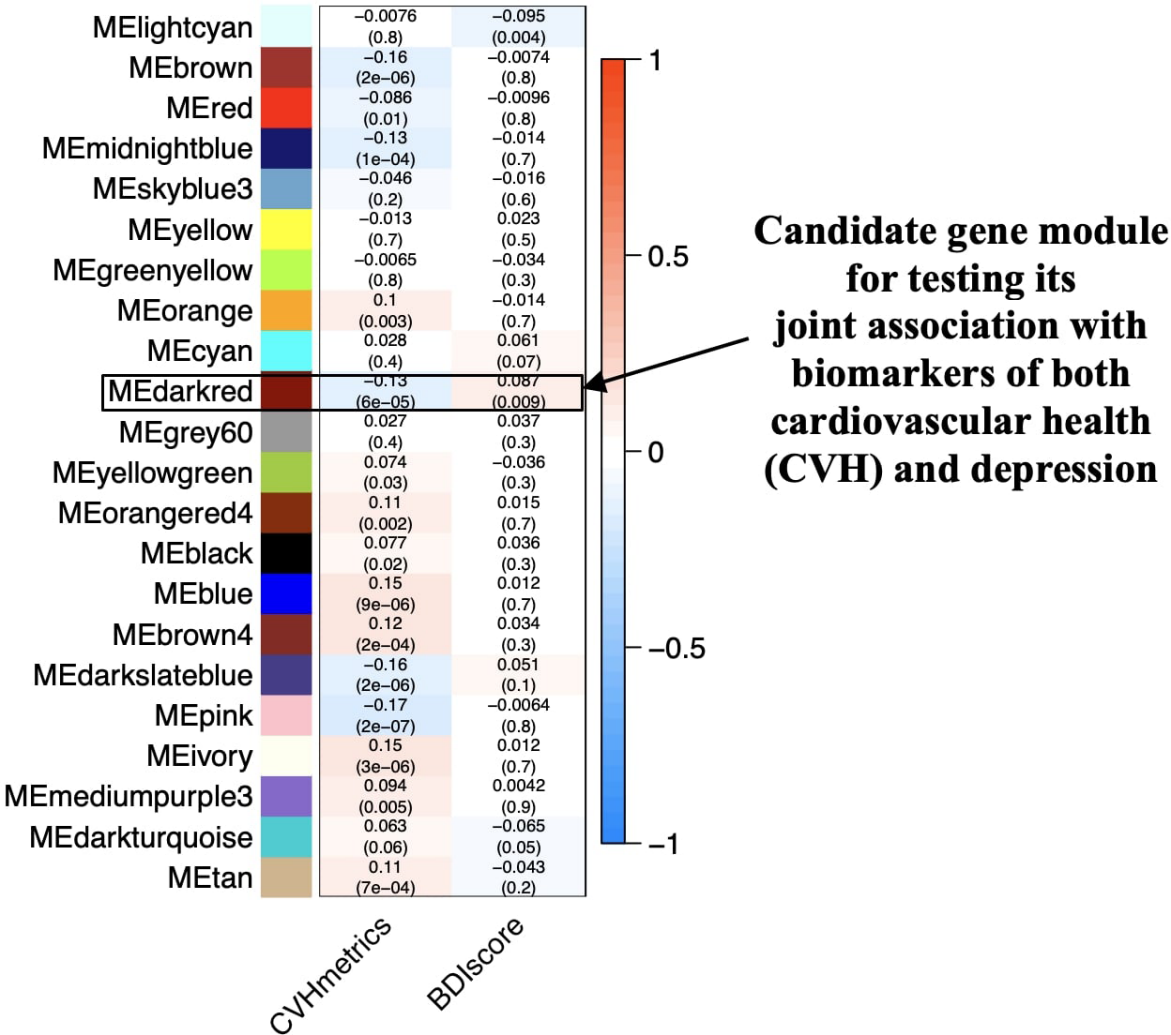
Relationships between gene co-expression modules (x-axis) and markers of cardiovascular health and depression (y-axis). The rows correspond to the different gene modules (named by color) and their summary expression profile; for example, MEdarkred represents the summary expression profile for the module darkred. The columns correspond to the markers of CVH (CVHmetrics) and depression (BDIscore) used in the biostatistical analyses. The values in the cells represent Pearson’s correlation coefficients (r), with the associated p-values in parentheses below the coefficient. The correlation coefficients have a color-coding shown in the color scale (between  −1 and + 1) on the right side of the figure. The highlighted darkred module is significantly associated with the markers of both CVH and depression. CVHmetrics, cardiovascular health metrics; BDIscore, Beck’s depression inventory score.

The dynamic tree-cutting algorithm identified 22 gene modules from the hierarchical clustering of the dissimilarity matrix, containing 14–10,367 highly correlated genes (**Figure 2**). Gene modules were named according to the colors assigned automatically by the *WGCNA* program. Among the 22 identified gene modules, 15 were associated with CVH metrics with a p-value <0.05, and only two were associated with the BDI-II score with the same p-value threshold. However, the gene module named darkred, containing 256 genes, was correlated with CVH metrics with a correlation coefficient (*r*) of −0.13, p-value of 6 × 10^−5^, and BDI-II score with *r* of 0.09 and *p*-value of 0.009. Therefore, as the darkred gene module was significantly correlated with markers of both CVH and depression, we considered it a candidate module that is potentially jointly associated with markers of both CVH and depression.

#### 3.4 Gene significance and module membership

**Figure 3:**
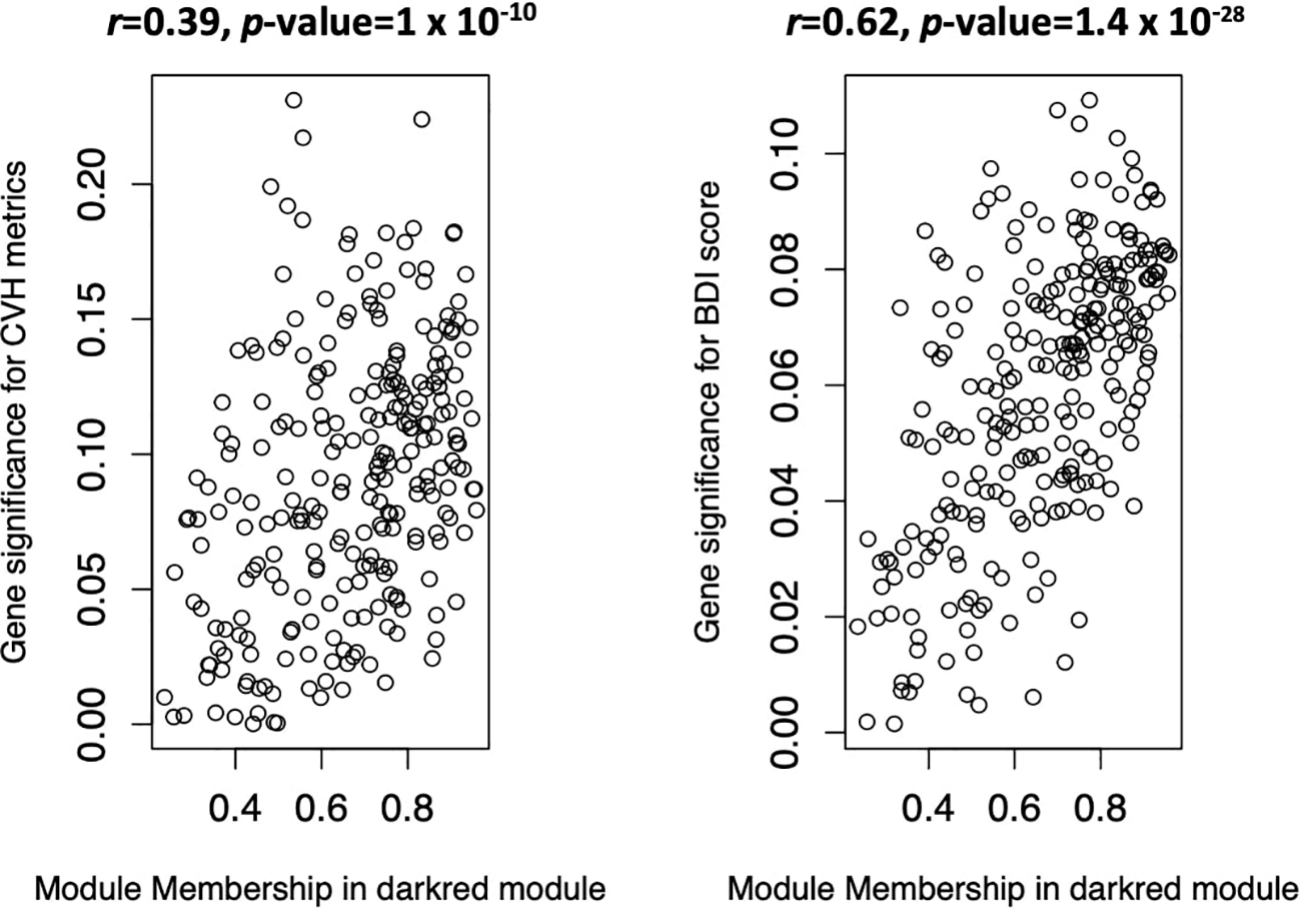
Scatter plots of gene significance (GS) vs module membership (MM) in the darkred gene module. The left panel corresponds to cardiovascular health (CVH) metrics and the right panel to Beck’s depression inventory score, markers of CVH and depression, respectively. CHV metrics, cardiovascular health metrics; BDI-II score, Beck’s depression inventory score.

GS is defined as the correlation between the module’s member genes and study markers. MM is defined as the correlation between an eigengene and other member genes of the module. An ideal gene module is one where GS and MM are highly correlated, suggesting that the genes that are highly correlated with the study markers are also important members of the gene module (27). The darkred gene module had a highly significant correlation between GS and MM with respect to both CVH metrics (*r* = 0.39, *p*-value = 1.0 × 10^−10^) and BDI-II score (*r* = 0.62, *p*-value = 1.4 × 10^−28^) (**Figure 3**).

#### 3.5 Multivariate analysis of the candidate gene module with the markers of cardiovascular health and depression

Based on the MANOVA test adjusted for age and sex of the participants, there was a statistically significant joint association between the eigengene of the darkred gene module and markers of both CVH and depression (Pillai’s Trace = 0.02, *p*-value = 1.8 × 10^−5^). The Pillai’s trace statistic ranges from 0 to 1, with higher values indicating a higher effect of the module’s summary expression profile on the study markers.

#### 3.6 Multivariate analysis of the member genes of the gene module jointly associated with the markers of cardiovascular health and depression

We also conducted a MANOVA test for the member genes of the dared-gene module to identify the most significant genes in the module. Ninety-five out of 256 genes had statistically significant joint associations with both CVH metrics and BDI-II scores (p.adj <0.05) (**Supplementary Table S1**). The 25 most significant member genes from the darkred gene module and their descriptions are presented in **Table 2**.

**Table 2:** List of the top 25 genes that are jointly associated with markers of both cardiovascular health and depression with Bonferroni adjusted *p*-value <0.05.

Genes	Full names	Pillai’s trace statistics	*p*-values	Bonferroni adjusted *p*-values
YOD1	YOD1 deubiquitinase	0.06	1.5 × 10^−12^	4.0 × 10^−10^
RBX1	ring-box protein 1	0.06	4.8 × 10^−12^	1.2 × 10^−09^
LEPR	leptin receptor	0.05	5.1 × 10^−10^	1.3 × 10^−07^
OPTN	optineurin	0.05	9.0 × 10^−10^	2.3 × 10^−07^
ATP5MJ	ATP Synthase Membrane Subunit J	0.04	1.2 × 10^−09^	3.0 × 10^−07^
S100A8	S100 calcium-binding protein A8	0.04	4.1 × 10^−09^	1.1 × 10^−06^
COX7B	cytochrome c oxidase subunit 7b	0.04	1.7 × 10^−08^	4.3 × 10^−06^
NDUFA4	NDUFA4 mitochondrial complex associated	0.04	1.8 × 10^−08^	4.6 × 10^−06^
SNORD3A	small nucleolar RNA, C/D box 3a	0.04	2.5 × 10^−08^	6.4 × 10^−06^
COX7C	cytochrome c oxidase subunit 7C	0.04	3.2 × 10^−08^	8.2 × 10^−06^
TMEM256	transmembrane protein 256	0.04	3.3 × 10^−08^	8.3 × 10^−06^
TP53RK	TP53 regulating kinase	0.04	6.5 × 10^−08^	1.7 × 10^−05^
LOC651202*	–	0.04	1.0 × 10^−07^	2.7 × 10^−05^
NDUFB3	NADH:ubiquinone oxidoreductase subunit B3	0.03	1.9 × 10^−07^	4.7 × 10^−05^
RPS15A	ribosomal protein S15a	0.03	2.0 × 10^−07^	5.1 × 10^−05^
ISCA1	iron-sulfur cluster assembly 1	0.03	2.1 × 10^−07^	5.3 × 10^−05^
SNHG29	small nucleolar RNA host gene 29	0.03	3.2 × 10^−07^	8.3 × 10^−05^
RPL39	ribosomal protein L39	0.03	3.5 × 10^−07^	8.9 × 10^−05^
LY96	lymphocyte antigen 96	0.03	3.5 × 10^−07^	9.1 × 10^−05^
CKS2	CDC28 protein kinase regulatory subunit 2	0.03	4.1 × 10^−07^	1.0 × 10^−04^
LOC100131387*		0.03	5.1 × 10^−07^	1.0 × 10^−04^
PSMA6	proteasome 20S subunit alpha 6	0.03	7.5 × 10^−07^	2.0 × 10^−04^
CEBPG	CCAAT enhancer binding protein gamma	0.03	1.0 × 10^−06^	3.0 × 10^−04^
LOC648622*	–	0.03	1.0 × 10^−06^	3.0 × 10^−04^
LOC644315 *	–	0.03	1.2 × 10^−06^	3.0 × 10^−04^

*Genes with their names beginning with LOC do not have a published symbol available and their orthologs determined.

#### 3.7 Pathway analysis of the gene module jointly associated with the markers of cardiovascular health and depression

**Figure 4:**
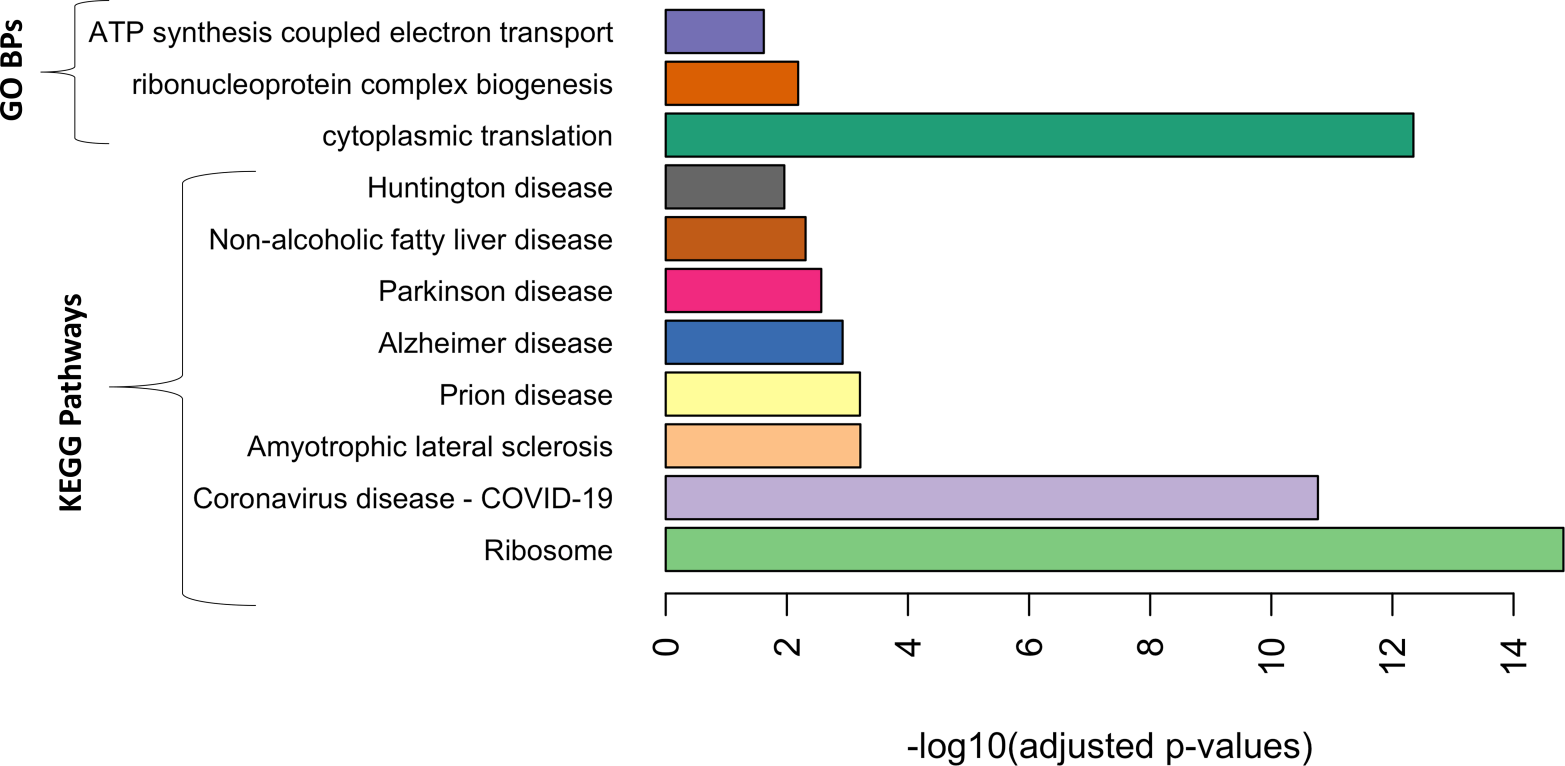
Gene Ontology biological processes (GO BPs) and Kyoto Encyclopedia of Genes and Genomes (KEGG) databases that were significantly enriched in the genes of the darkred gene module with adjusted *p*-value <0.05.

Genes in the darkred module were enriched with seven biological pathways from the KEGG database and five biological processes from the GO database (p.adj <0.05) (**Figure 4**).

## 4 Discussion

We performed a system-level analysis of whole blood transcriptomic data to identify gene modules and enriched biological pathways shared by markers of depression and CVH. We identified a shared gene module that was significantly associated with markers of both depression (BDI-II score) and cardiovascular health (CVH metrics). The biological pathways enriched among the members of the module were related to several mental disorders, ATP synthesis, and protein translation.

Accumulating evidence supports the idea that depression and CVD are co/multimorbid conditions (40). Interestingly, the relationship between these two conditions is bidirectional. For example, after adjusting for all known cardiovascular risk factors in studies of sizable populations free of medical conditions, people with baseline depression were more likely to develop cardiovascular disease (41, 42). Among people who develop coronary artery disease, those with depression, even a milder form, have a significantly higher risk of cardiac morbidity and mortality. Therefore, an important question related to CVD–depression co/multimorbidity is whether addressing depression can lower the risk of developing CVD or vice versa. An in-depth understanding of the underlying molecular mechanisms of the two conditions is of paramount importance to address this question.

The biological processes and pathways enriched in the darkred gene module, identified to be jointly associated with the early markers of both diseases, have been shown in previous studies to be associated with both diseases. For example, genes involved in GO biological process, cytoplasmic translation, and KEGG pathway ribosomes play a role in protein translation. Impairment of protein translation is associated with mental disorders, including major depression (43). Similarly, GO biological process, *ATP* synthesis coupled electron transport was also enriched among the member genes of the identified module, which is known to regulate depressive-like behavior (44). ATP depletion also plays a role in atherosclerosis (45). Most other pathways enriched in the identified gene module were related to neurodegenerative diseases, such as Huntington’s disease, Parkinson’s disease, and Alzheimer’s disease. The prevalence of depression among patients with neurodegenerative diseases is high (46). A pathway related to non-alcoholic fatty liver disease (NAFLD) was also enriched in this module. Interestingly, a recent study showed a significantly high prevalence of depression among patients with NAFLD (47). NAFLD is an independent risk factor for atherosclerotic CVD (48).

The top three genes of the module, *YOD1*, *RBX1*, and *LEPR*, have been shown previously to be associated with neurodegenerative diseases, bipolar disorder, and depression (49–51). *YOD1* belongs to the ubiquitin–proteasome system and plays an important role in protein metabolism. Deubiquitinases stabilize the expression of their target proteins by removing their ubiquitin chains. Alterations in the ubiquitin–proteasome system can lead to inflammation, neurodegeneration and cancer (52). Deubiquitinases are involved in cell death and inflammation (53), and both play major roles in atherosclerosis (54). Similarly, *RBX1* is a component of the ubiquitin–proteasome system and is known to play a role in inflammation and neurodegeneration (52). Inflammation is associated with diabetes, obesity, and hypertension (55–57). We speculate that the significant association between the genes and CVH metrics observed in this study was driven, at least partly, by blood glucose level, body mass index, and blood pressure, which are three of the components of the CVH metrics. Therefore, this study uncovered potential genes that may explain the inflammation-based link between depression and CVH. The *LEPR* protein is present on the cell surfaces of numerous organs and tissues, including the hypothalamus, and regulates body temperature, sleep, mood, hunger, and thirst. *LEPR* is activated by the adipokine hormone leptin, which is generated and secreted by the white adipose tissue. The attachment of leptin to its receptor in the hypothalamus sets off a cascade of chemical signals that influence hunger and contribute to a sensation of fullness. Neural plasticity, a mechanism of neuronal adaption, is affected by leptin. The onset and progression of depression are influenced by modifications in neural plasticity caused by stress and other adverse stimuli (58). Elevated leptin levels are a major risk factor for atherosclerotic CVD. Leptin exerts proatherogenic effects on a variety of vascular cell types, including macrophages, endothelial cells, and smooth muscle cells via its interaction with LEPR, which is highly expressed in atherosclerotic plaques (59). To the best of our knowledge, this is the first study to identify the role of *LEPR* in the pathogenesis of both depression and atherosclerotic CVD. A similar transcriptomic study was recently published (60). However, unlike the study by (60), the unique feature of this study is that we focused on the identification of transcriptomic signatures that were jointly associated with markers of both depression and CVH among the same set of participants.

We speculate that the statistically significant joint association of the genes with depression and CVH metrics observed in this study could be driven by one or both of the following scenarios: i) unhealthy behavior-related components of the CVH metrics such as smoking, unhealthy diet, and lack of physical activity lead to altered expression levels of the genes, which in turn stimulate biological pathways leading to depression; ii) the genes stimulate biological pathways leading to both depression as well as the health factor-related components of the CVH metrics such as total blood cholesterol level, blood pressure, and blood glucose level. We believe that the associations observed in this study can stimulate further research for deeper understanding of the depression–CVD co/multimorbid. For instance, establishing whether the observed associations of the gene network and the genes with the markers of mental and cardiovascular health are causal requires further research involving clinical CVD and depression outcomes in multiethnic cohorts.

This study has several limitations. One limitation was the lack of a clinical CVD endpoint, as the study was based on a relatively young population cohort with an early phase of cardiovascular disease. Instead, CVH metrics have been shown to be associated with subclinical atherosclerosis (61). This study used microarray technology because, at the time of the follow-up (year 2011), RNA-Seq technology was still too expensive for a sizable epidemiological study like the one in this study. Additionally, all the participants in the study were of European descent. Therefore, further research with populations of different ethnicities, age groups, and clinical CVD endpoints is required.

It is imperative to consider depression and cardiovascular disease co/multimorbidity for the best possible care and improved clinical outcomes. However, as the current healthcare systems around the world are built to concentrate on treating specific diseases, the holistic care of individuals with both conditions is challenging. A comprehensive understanding of the joint risk factors, clinical characteristics, and molecular networks underlying these conditions is necessary to develop a holistic treatment approach for patients with these conditions, for example, by identifying key molecules such as genes that can be targeted for intervention, considering their effect on both conditions and potential side effects. The availability of high-throughput biological datasets, such as omics and sophisticated machine learning-based data science methods, makes it feasible to uncover and develop a comprehensive and shared landscape of the molecular networks underlying these conditions. This study aids in transitioning the focus of contemporary biomedical research from a single-disease framework to a spectrum of multiple diseases that may coexist and have a shared biological base.

Overall, our results from the transcriptome-wide multivariate association analysis of mental and cardiovascular health markers support the depression and CVD co/multimorbidity hypothesis. This study identified several genes and biological pathways jointly associated with markers of both conditions, shedding light on the potential molecular mechanisms underlying these two related conditions. The identified gene module and its most significant member genes can provide new joint biomarkers and therapeutic targets for depression and CVH after validation in further studies. Joint biomarkers may facilitate the development of dual-purpose preventative strategies and help predict co/multimorbid disorders. Additionally, this study highlights a system-level multivariate biostatistical approach for analyzing omics data in co/multimorbidity studies.

## Data availability statement

The dataset supporting the conclusions of this article were obtained from the Cardiovascular Risk in Young Finns study which comprises health related participant data. The use of data is restricted under the regulations on professional secrecy (Act on the Openness of Government Activities, 612/1999) and on sensitive personal data (Personal Data Act, 523/1999, implementing the EU data protection directive 95/46/EC). Due to these restrictions, the data cannot be stored in public repositories or otherwise made publicly available. Data access may be permitted on a case-by-case basis upon request only. Data sharing outside the group is done in collaboration with YFS group and requires a data-sharing agreement. Investigators can submit an expression of interest to the chairman of the publication committee, Prof OR (Turku University, Finland), Prof MK (Tampere University, Finland) and Prof TL (Tampere University, Finland). Requests to access these datasets should be directed to OR, olli.raitakari@utu.fi; TL, terho.lehtimaki@tuni.fi; MK, mika.kahonen@tuni.fi.

## Ethics statement

The studies involving humans were approved by the ethical committee of the Hospital District of Southwest Finland on 20 June 2017 (ETMK:68/1801/2017). The studies were conducted in accordance with the local legislation and institutional requirements. The participants provided their written informed consent to participate in this study.

## Author contributions

BHM: Conceptualization, Formal analysis, Investigation, Methodology, Writing – original draft. ER: Writing – review & editing. NM: Writing – review & editing. AS: Writing – review & editing. JV: Writing – review & editing. MJ: Writing – review & editing. NHK: Writing – review & editing. MK: Writing – review & editing. OTR: Resources, Writing – review & editing. TL: Resources, Writing – review & editing. PPM: Conceptualization, Formal analysis, Investigation, Methodology, Writing – original draft.
