# Supplementary material for: Identification of gene networks jointly associated with depressive symptoms and cardiovascular health metrics using whole blood transcriptome in the Young Finns Study
Source: Front Psychiatry. 2024 Apr 25;15:1345159. doi: 10.3389/fpsyt.2024.1345159 (PMC11079127; doi:10.3389/fpsyt.2024.1345159)
Supplement: Supplementary file 1 [file DataSheet_1.pdf]

Identification of gene networks jointly associated with depressive symptoms and cardiovascular health metrics using whole blood transcriptome in the Young Finns Study

Binisha H. Mishra[1,2,3], Emma Raitoharju[4,5], Nina Mononen[1,2,3], Aino Saarinen[6,7], Jorma Viikari[8,9], Markus Juonala[8,9], Nina Hutri-Kähönen[10], Mika Kähönen[2,11], Olli T. Raitakari[12,13,14], Terho Lehtimäki[1,2,3], Pashupati P. Mishra[1,2,3]

[1]Department of Clinical Chemistry, Faculty of Medicine and Health Technology, Tampere University, Tampere, Finland
[2]Finnish Cardiovascular Research Center Tampere, Faculty of Medicine and Health Technology, Tampere University, Tampere, Finland
[3]Department of Clinical Chemistry, Fimlab Laboratories, Tampere, Finland
[4]Molecular Epidemiology, Faculty of Medicine and Health Technology, Tampere University, Tampere, Finland
[5]Tampere University Hospital, Tampere, Finland
[6]Department of Psychology and Logopedics, Faculty of Medicine, University of Helsinki, Finland
[7]Helsinki University Central Hospital, Adolescent Psychiatry Outpatient Clinic, Helsinki, Finland
[8]Department of Medicine, University of Turku, Turku, Finland
[9]Division of Medicine, Turku University Hospital, Turku, Finland
[10]Department of Paediatrics, Tampere University Hospital, Faculty of Medicine and Health Technology, Tampere University, Tampere, Finland
[11]Department of Clinical Physiology, Tampere University Hospital, Tampere Finland.
[12]Research centre of Applied and Preventive Cardiovascular Medicine, University of Turku, Turku, Finland
[13]Department of Clinical Physiology and Nuclear Medicine, Turku University Hospital, Turku, Finland
[14]Centre for Population Health Research, University of Turku and Turku University Hospital, Turku, Finland

***Correspondence to:**

Binisha H. Mishra (binisha.hamalmishra@tuni.fi)

Faculty of Medicine and Health Technology, Tampere University, Tampere 33520, Finland, and Department of Clinical Chemistry, Fimlab Laboratories, and Finnish Cardiovascular Research Center – Tampere

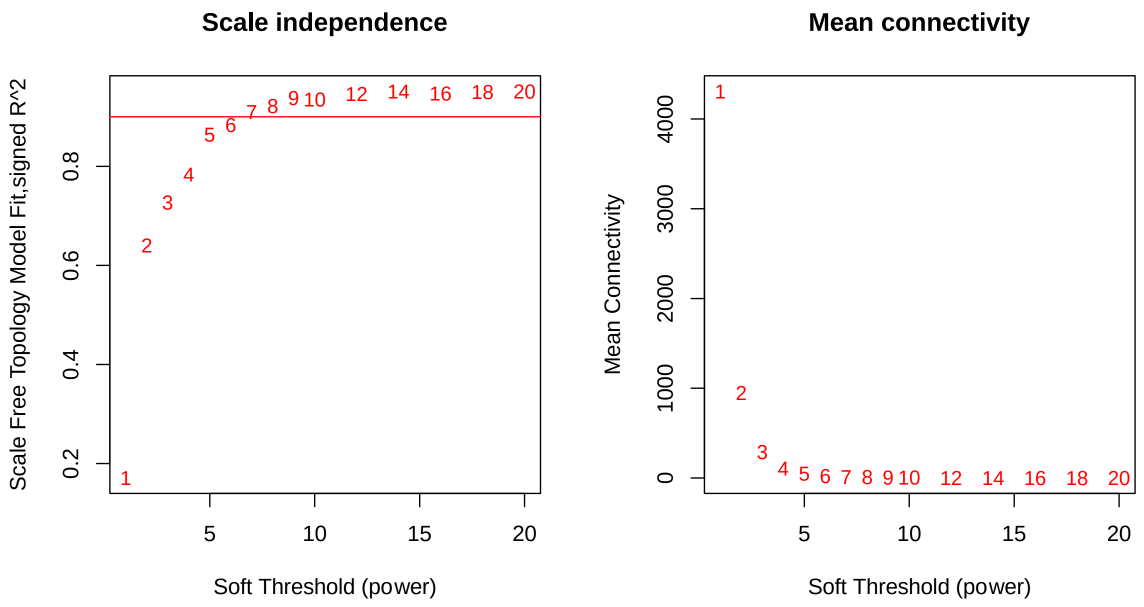

Figure S1. Determination of soft-thresholding power in weighted gene co-expression network analysis. Summary network indices (scale free topology and mean connectivity) (y-axis) as functions of the soft thresholding power (x-axis). The plot indicates that approximate scale-free topology is attained around the soft-thresholding power of 5.

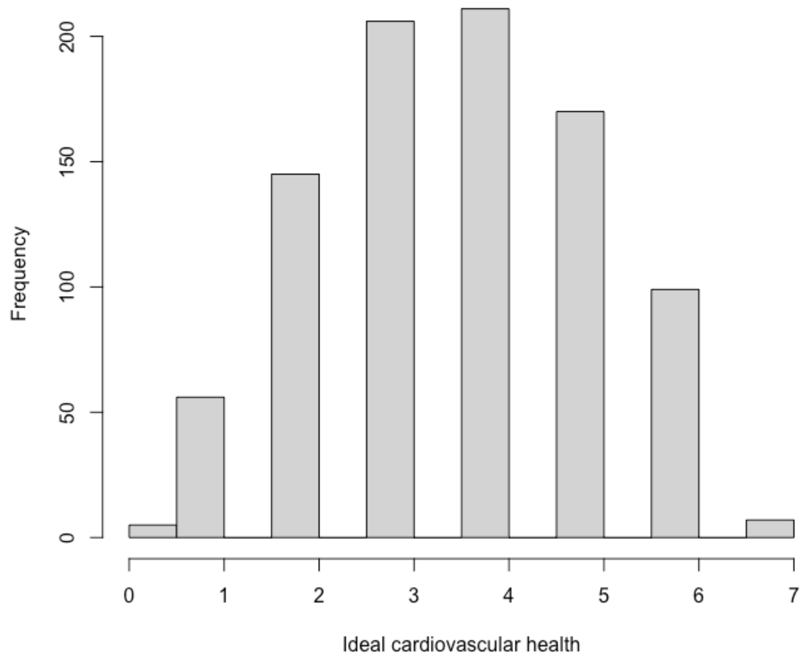

Figure S2: Distribution of cardiovascular health (CVH) metrics among the Young Finns Study (YFS) participants.

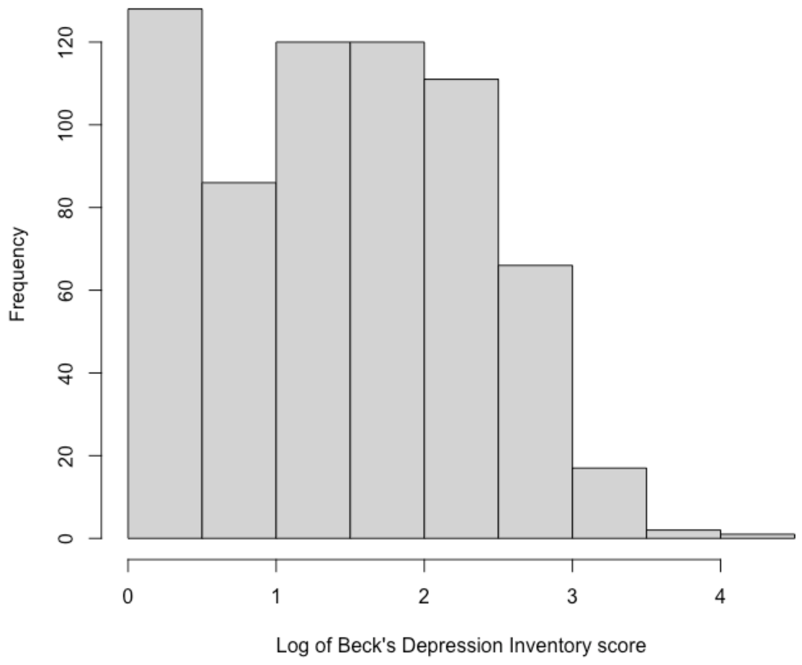

Figure S3: Distribution of Beck’s depression inventory (BDI-II) scores among the Young Finns Study (YFS) participants.

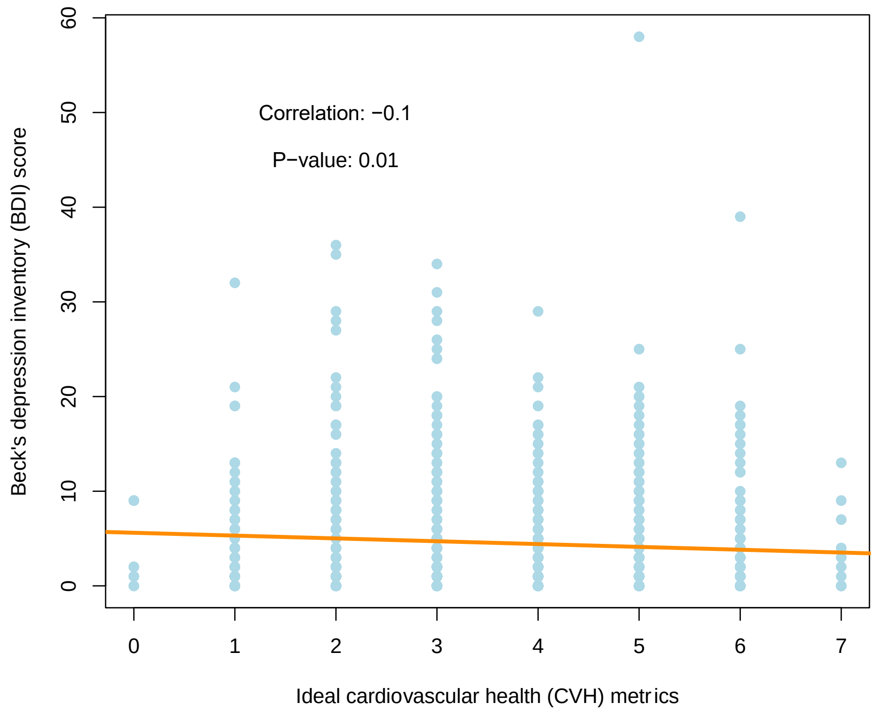

Figure S4: Correlation between the Beck’s depression inventory (BDI-II) scores and ideal cardiovascular health (CVH) metrics among the Young Finns Study (YFS) participants.
